# Correction: Sun et al. New Non-Fullerene Acceptor with Extended Conjugation of Cyclopenta [2,1-b:3,4-b’] Dithiophene for Organic Solar Cells. *Molecules* 2022, *27*, 7615

**DOI:** 10.3390/molecules31050899

**Published:** 2026-03-09

**Authors:** Cheng Sun, Sanseong Lee, Changeun Choi, Soyeong Jeong, Juhui Oh, Ju-Hyeon Kim, Jaeyoung Kim, Ho Eon Baek, Hongkyu Kang, Soo-Young Jang, Hyun Ho Choi, Kwanghee Lee, Yun-Hi Kim

**Affiliations:** 1 Department of Chemistry and RIGET, Gyeongsang National University, Jinju 52828, Republic of Korea; suncheng@qibebt.ac.cn (C.S.); qpqrryytwie@gmail.com (H.E.B.); 2 School of Materials Science and Engineering, Gwangju Institute of Science and Technology, Gwangju 61005, Republic of Korea; sanseong@gm.gist.ac.kr (S.L.); jumi0504@gm.gist.ac.kr (J.O.); juhyeon.kim@liu.se (J.-H.K.); jyk941231@naver.com (J.K.); 3 Heeger Center for Advanced Materials, Research Institute for Solar and Sustainable Energies, Gwangju Institute of Science and Technology, Gwangju 61005, Republic of Korea; sowh013@naver.com (S.J.); 4 Department of Materials Engineering and Convergence Technology and RIGET, Gyeongsang National University, Jinju 52828, Republic of Korea; dusrnr3579@naver.com; 5 Center for Research Innovation, Gwangju Institute of Science and Technology, Gwangju 61005, Republic of Korea; gemk@gist.ac.kr; 6 Research Institute for Solar and Sustainable Energies, Gwangju Institute of Science and Technology, Gwangju 61005, Republic of Korea

## Text Correction

In the original publication [1], the co-first authorship for Sanseong Lee was unintentionally omitted due to an administrative error. To ensure the scholarly record accurately reflects the equal contributions of the primary researchers, the authors wish to revise the ‘Author Contributions’ section to formally recognize the co-first authorship and provide a more detailed account of each author’s role.

Author Contributions: C.S. and S.L. contributed equally to this work. Conceptualization: C.S., S.L., K.L.; Methodology: C.S., S.L.; Investigation: C.S., S.L., S.J.; Formal analysis: C.S., S.L., C.C., S.J., J.O., J.-H.K., J.K., H.E.B.; Data curation: H.K., H.H.C.; Validation: H.K.; Visualization: S.L., J.K.; Writing—original draft preparation: C.S., S.L., J.K., S.-Y.J.; Writing—review and editing: S.-Y.J., H.H.C.; Supervision: S.-Y.J., K.L., Y.-H.K.; Project administration: S.-Y.J., H.H.C., K.L., Y.-H.K.; Funding acquisition: K.L., Y.-H.K. All authors have read and agreed to the published version of the manuscript.

The authors state that the scientific conclusions are unaffected. This correction was approved by the Academic Editor. The original publication has also been updated.
