# Supplementary material for: New Non-Fullerene Acceptor with Extended Conjugation of Cyclopenta [2,1-b:3,4-b’] Dithiophene for Organic Solar Cells
Source: Molecules. 2022 Nov 6;27(21):7615. doi: 10.3390/molecules27217615 (PMC9658291; doi:10.3390/molecules27217615)
Supplement: Supplementary file 1 [file molecules-27-07615-s001.zip › molecules-2023305-supplementary.pdf]

1.Materials

All solvents, reagents, and other materials were used as received from common commercial level and purchased from Sigma-Aldrich, TCI chemicals, and Alfa Aesar. All solvents used were purified prior to use. INCN, INCN-Me, INCN-F and INCN-Cl were purchased from derthon optoelectronic materials sci. tech. co., ltd. Diethyl 2,5-bis(4,4-dimethyl-4H-cyclopenta[2,1-b:3,4-b']dithiophen-2-yl)terephthalate (1), 1-bromo-3-((2-ethylhexyl)oxy)benzene were synthesized according to previous reports [50-51], respectively.

2. Measurements

^{1}H-NMR and ^{13}C-NMR spectra were measured on a Bruker DRX-300 and DRX-500 MHz spectrometer. Mass spectrometer, EI and FAB, were measured using a High-Resolution Mass Spectrometer, JEOL JMS-700. The thermal gravimetric analysis (TGA) was recorded with TA TGA 2100 thermogravimetric analyzer in a nitrogen atmosphere at a rate of 10 °C/min. Differential scanning calorimetry (DSC) was conducted under nitrogen on a TA instruments, 2100 DSC. Each sample was heated at 10 °C/min from 30 °C to 300 °C. The UV-Visible absorption spectra were obtained with a Perkin-Elmer LAMBDA-900 UV-vis/IR. The cyclic voltammetry (CV) was performed on an EG and G Parc model 273 Å potentiostat/galvanostat system with a three-electrode cell in a solution of 0.1M tetrabutylammonium perchlorate (Bu_4NClO_4) in chloroform at a scan rate of 50 mVs^{-1}. A glassy carbon electrode was used as the working electrode. An $Ag/AgNO_3$ electrode was used as the reference electrode and a Pt wire was used as the counter electrode. The J−V characteristics of the OPV devices were obtained using a Keithley 2420 with simulated AM 1.5 illumination (100 mW cm−2) by a solar simulator (Oriel Sol3A 450W, Newport). The external quantum efficiency (EQE) spectrums were measured using ORIEL QuantX 300 (Newport). The QuantX 300 equipment were calibrated using a silicon detector

calibrated by Newport. The out-of-plane X-ray diffraction profiles were attained using wide-angle X-ray scattering (WAXS) on the 3D high resolution X-ray diffractometer (EMPyrean, Malvern Panalytical Ltd.) over the 2θ range from 3° to 9°. The AFM images of the NFA and blending films were obtained utilizing AFM instrument XE-100 (Park Systems, Inc.) in tapping mode.

3. Device Fabrication

Pre-patterned ITO-coated glass substrates were swapped with detergent, and then sonicated in deionized water, acetone and isopropyl alcohol for 10 min, respectively. Subsequently, they were dried in a convection oven for an hour. The ITO substrates were treated with UV-ozone for 30 min. ZnO precursor solution (0.8 g of zinc acetate dehydrate was dissolved in 50.9 ml of isopropyl alcohol, and then 0.4 g of ethanolamine was added in the solution) was dropped on the substrates and spin-coated at 3000 rpm for 30 s, annealed at 200 °C for 10 min. The PBDB-T:m-Me-ITIC (1:1.2), PBDB-T:m-Me-ITIC-Me (1:1.2), PBDB-T:m-Me-ITIC-F (1:1), and PBDB-T:m-Me-ITIC-Cl (1:1) blends were dissolved in chloroform solvent with a total solution concentration of 20 mg ml-1 and with 0.5 vol% 1-chloronaphthalene only for PBDB-T:m-Me-ITIC-F and 1.5 vol% 1,8-diiodoocatane only for PBDB-T:m-Me-ITIC-Cl as additives. The solutions were stirred for at least 3 h at 60 °C. After cooling down the solutions, the solutions spin-coated on the ZnO film at 2500 rpm for 30 s in N_2 glove box and pre-annealed at 160 °C for 20 min. Afterwards, a 5 nm Molybdenum oxide (MoO_3) and 100 nm Ag ware deposited by thermal evaporation in a vacuum (4 x 10-6 Torr). The device active area is 4.64 mm2.

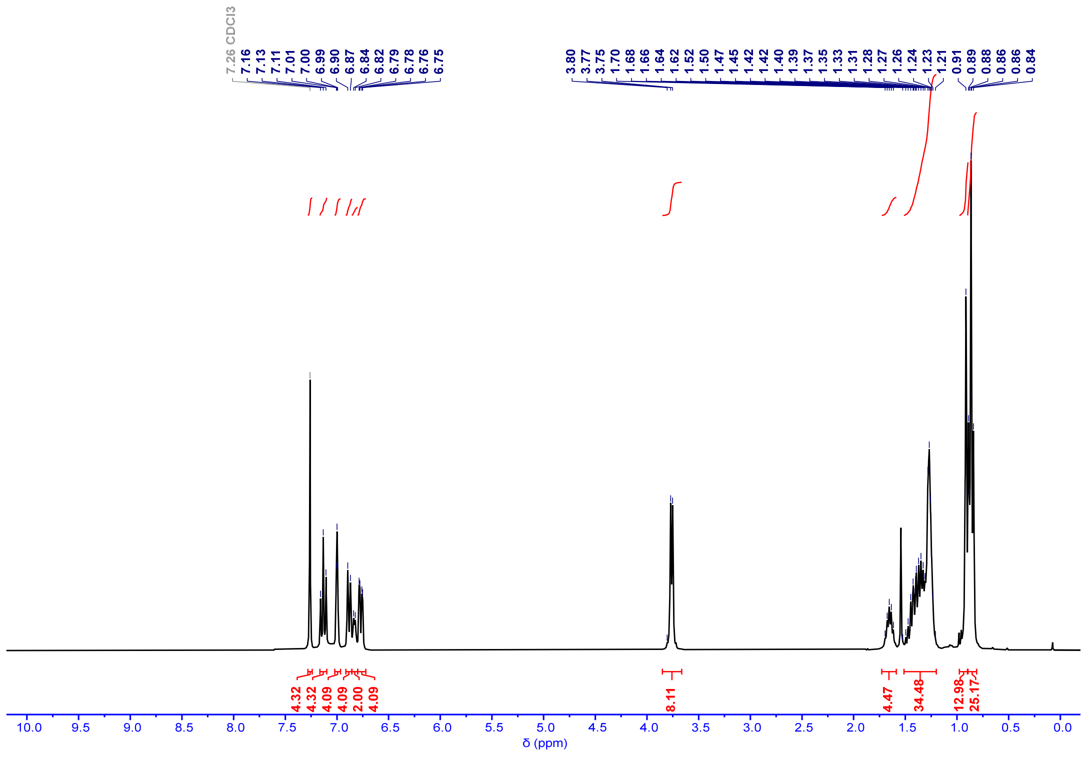

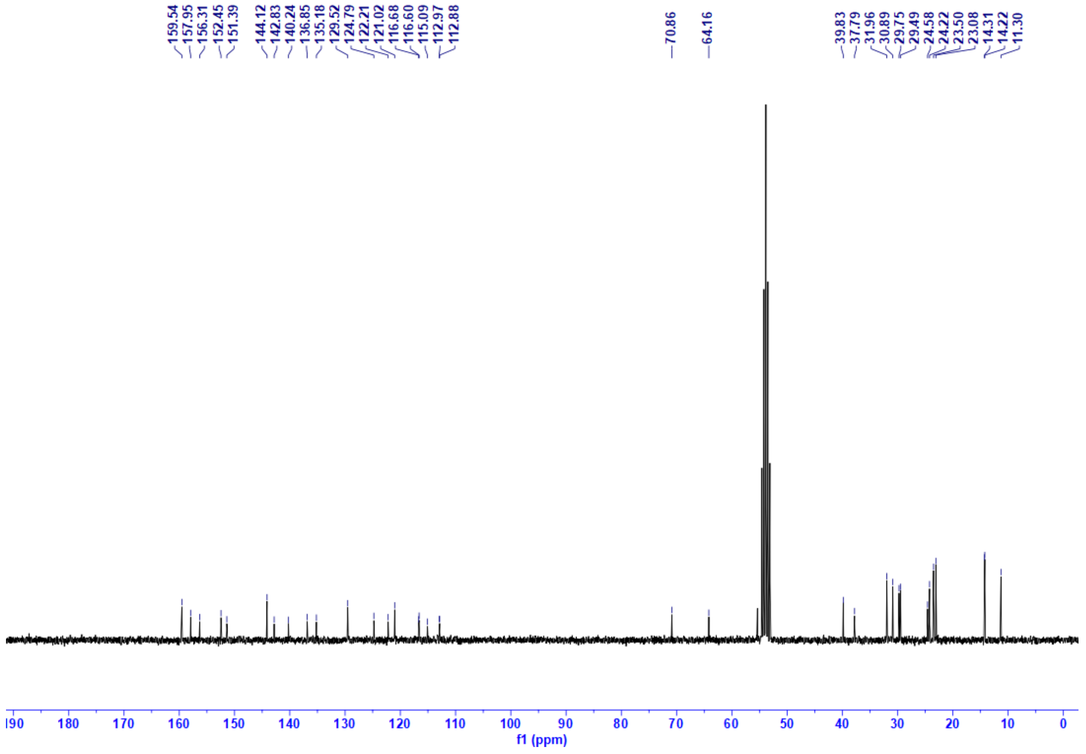

Figure S1. **^{1}H-NMR and ^{13}C-NMR of compound 2.**

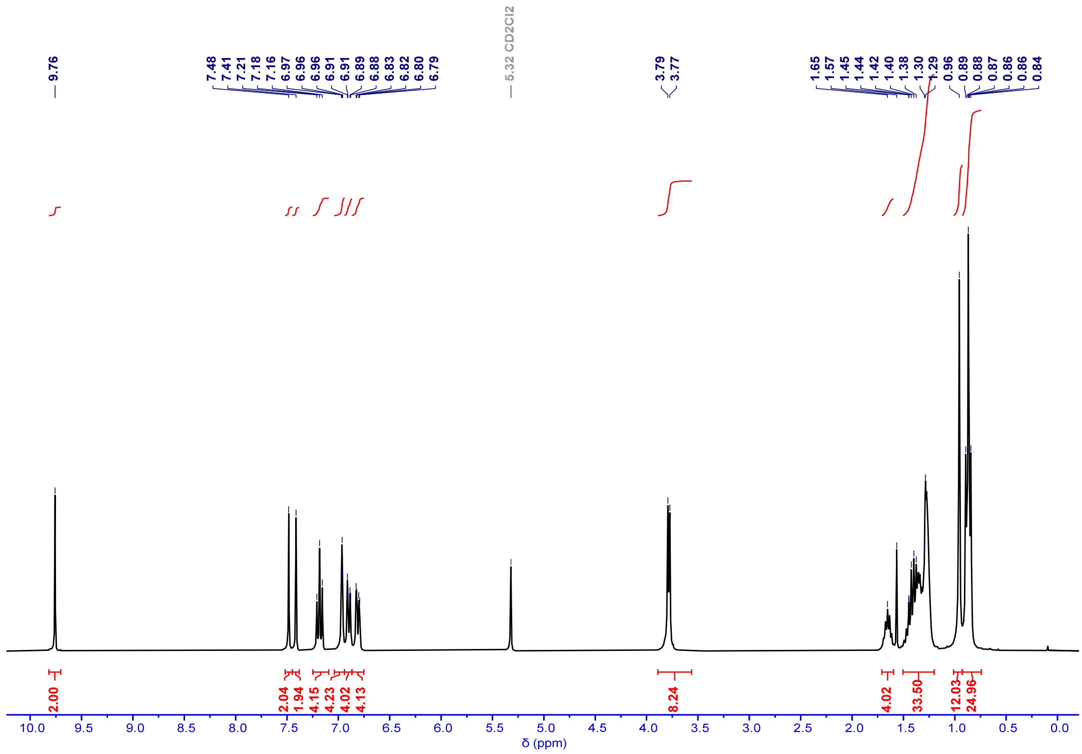

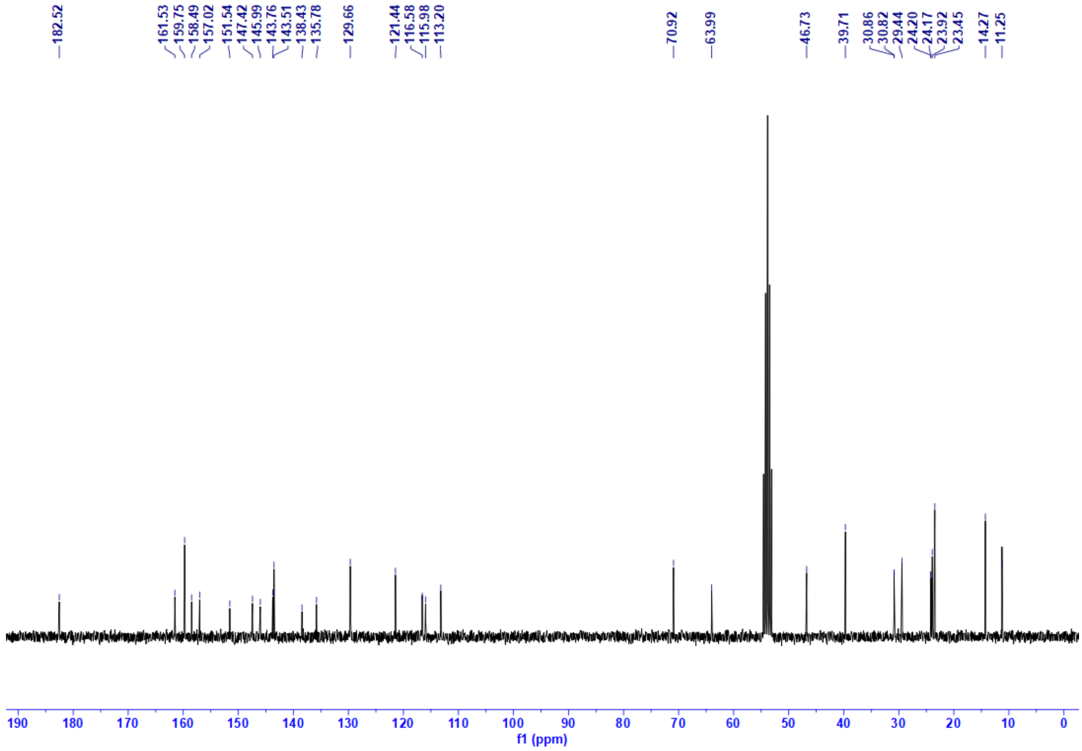

Figure S2. ^{1}**H-NMR and** ^{13}**C-NMR of compound 3.**

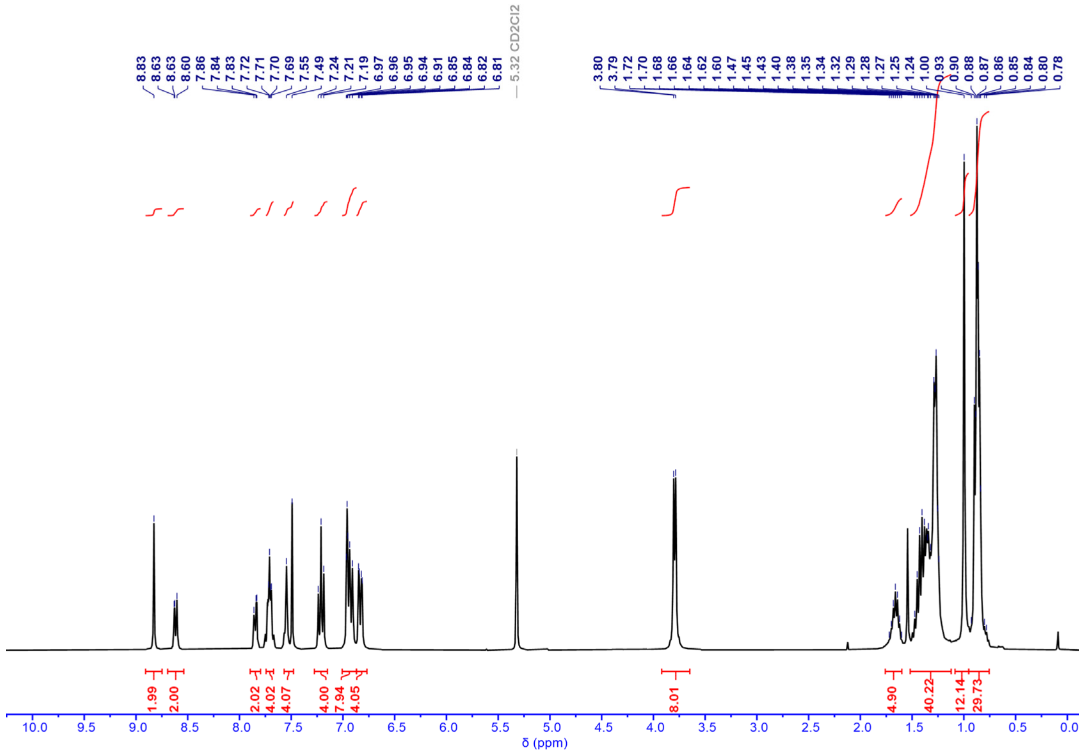

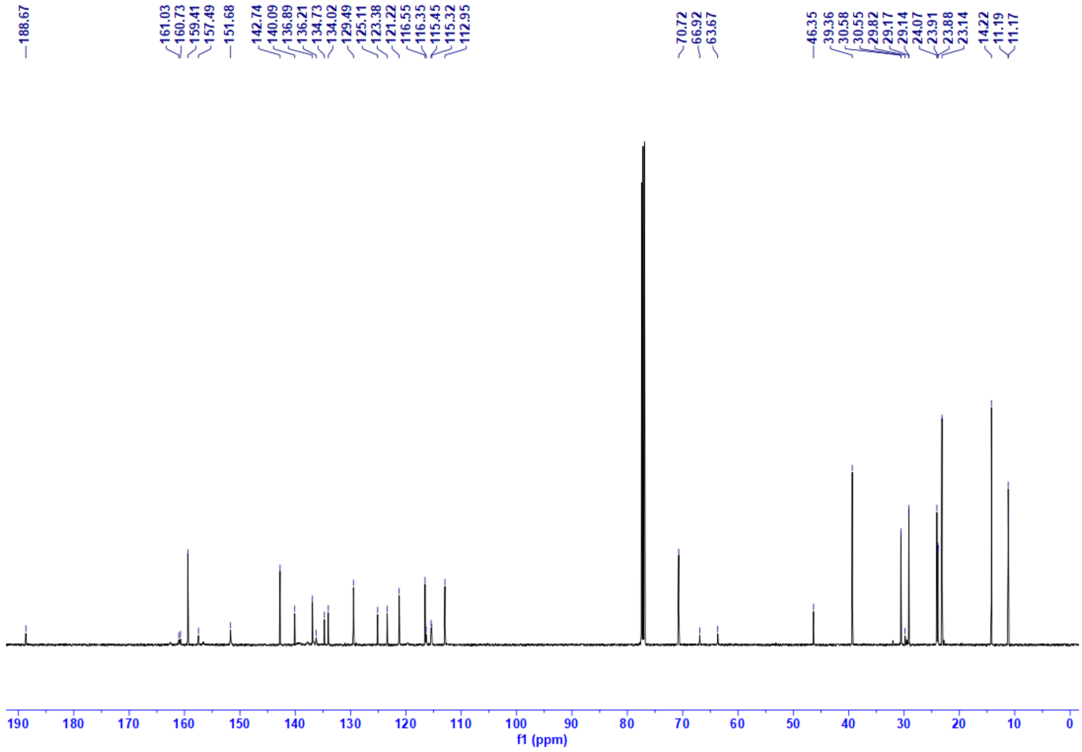

Figure S3. ^{1}H-NMR and ^{13}C-NMR of m-Me-ITIC.

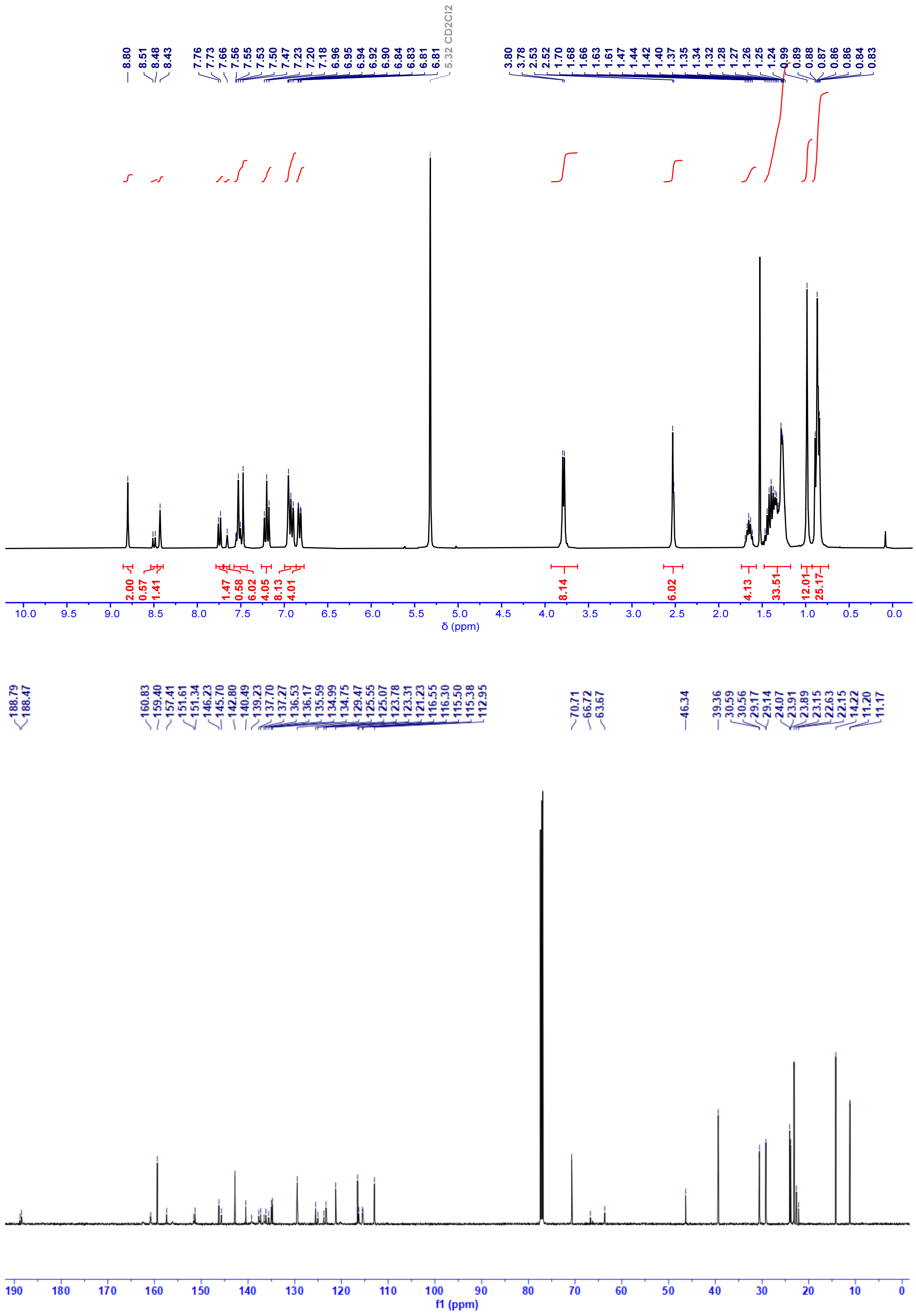

Figure S4. ^{1}H-NMR and ^{13}C-NMR of compound m-Me-ITIC-Me.

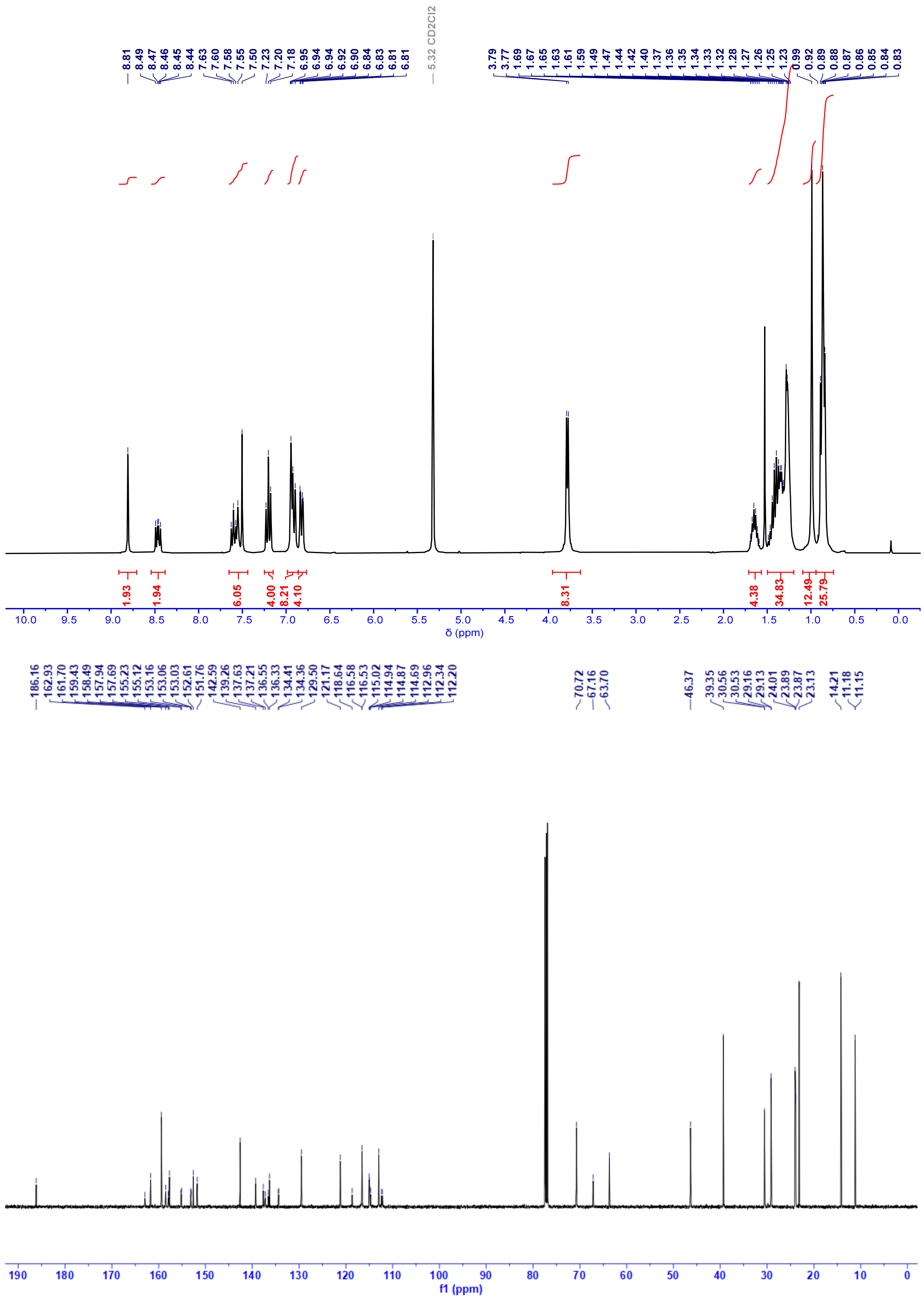

Figure S5. ^{1}H-NMR and ^{13}C-NMR of m-Me-ITIC-F.

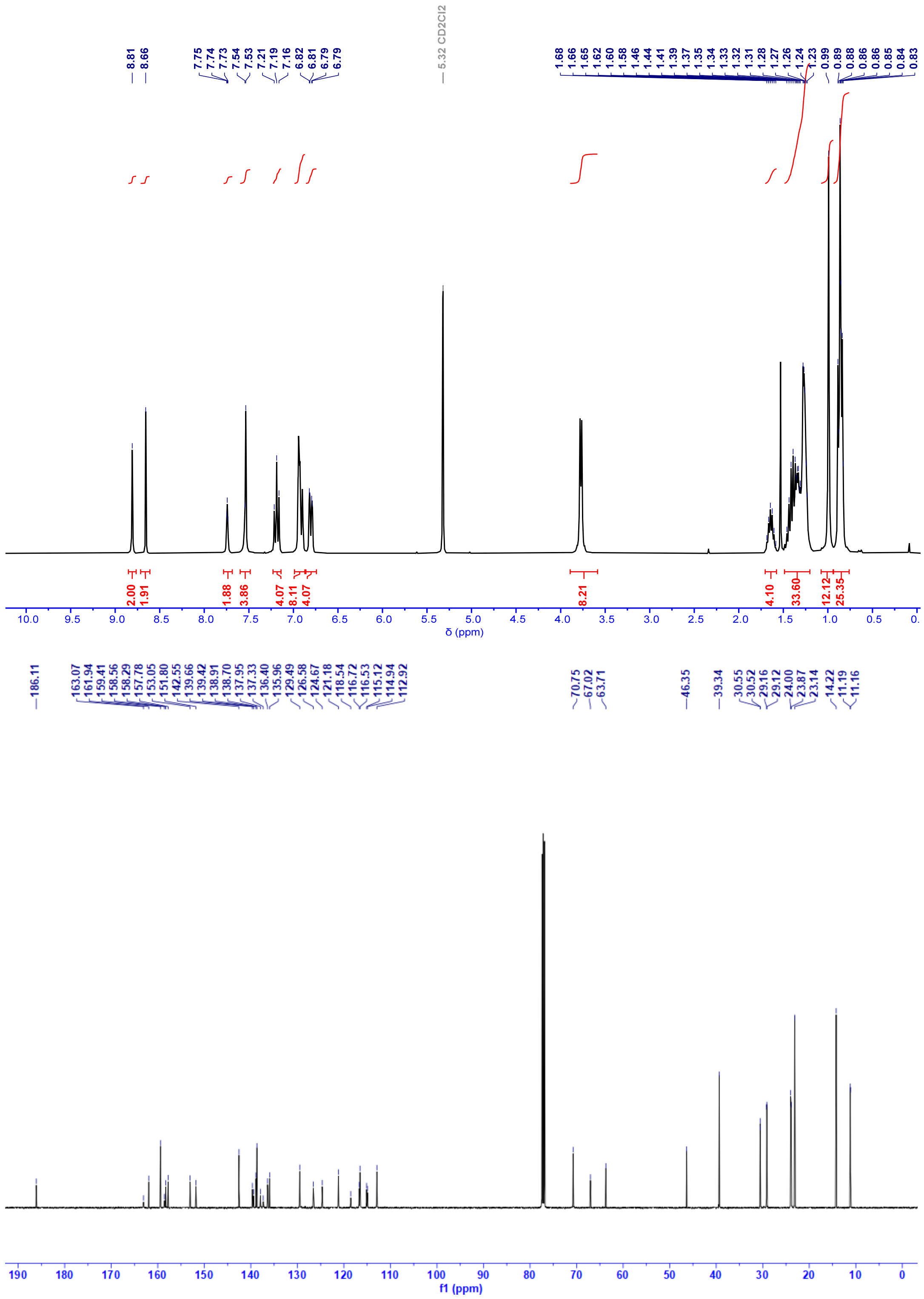

Figure S6. ^{1}H-NMR and ^{13}C-NMR of m-Me-ITIC-Cl.

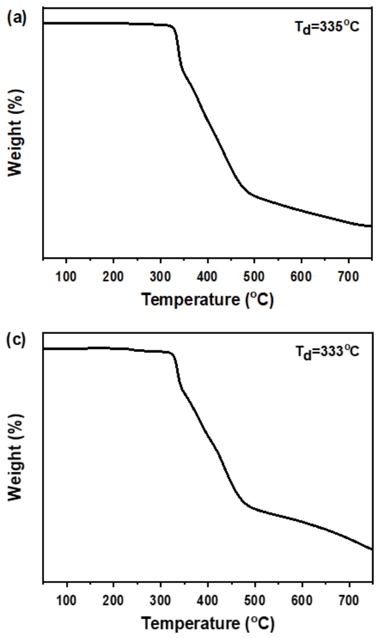

Figure S7. TGA curves of (a) m-Me-ITIC, (b)m-Me-ITIC-Me, (c) m-Me-ITIC-F, and (d)m-Me-ITIC-Cl with a heating rate of 10 °C/min.

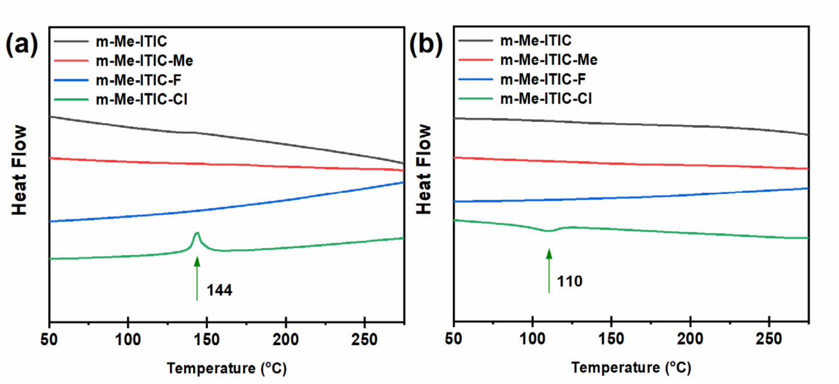

Figure S8. DSC thermograms of m-Me-ITIC-X (a) heat-only thermograms and (b) cool-only thermograms.

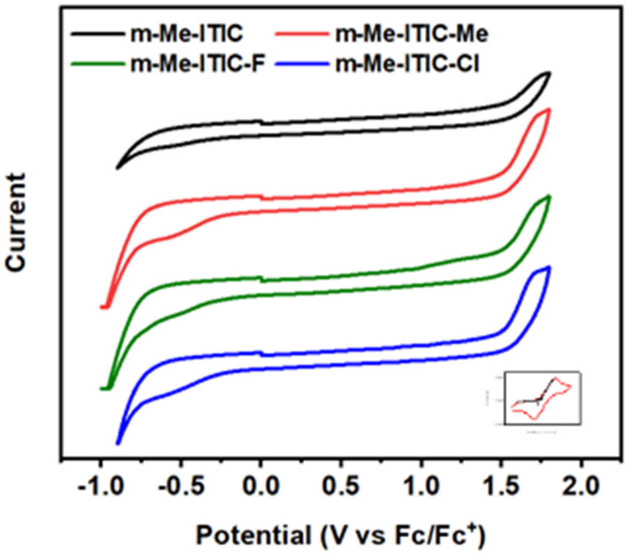

Figure S9. CV curves measured from Chloroform solution of m-Me-ITIC-Xs.

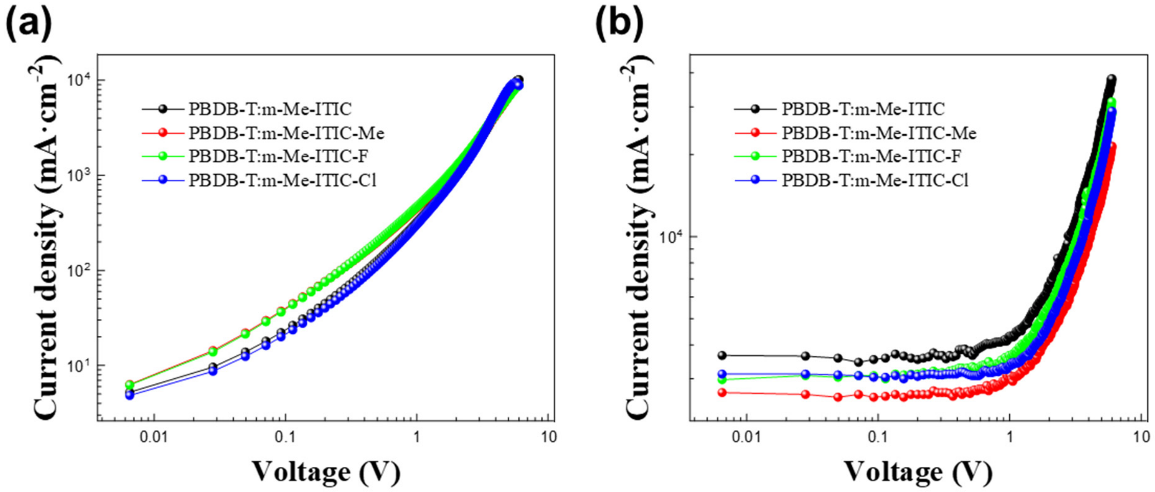

Figure S10. SCLC measurements of hole- and electron-only devices. J-V characteristics of (a) hole-only devices and (b) electron-only devices based on PBDB-T and the acceptors blend films.

Table S1. The photovoltaic parameters of the OPVs based on PBDB-T:m-Me-ITIC, PBDB-T:m-Me-ITIC-Me, PBDB-T:m-Me-ITIC-F, and PBDB-T:m-Me-ITIC-Cl with or without thermal annealing for 20min under the illumination of AM 1.5G, 100 mW·cm^{-2}.

Photoactive materials	Annealing (°C)	V_{OC} (V)	J_{SC} (mA·m^2)	EQE (mA·cm^2)	FF	PCE[a] (%)
PBDB-T:m-Me-ITIC	-	0.93	7.4	8.82	0.39	2.68
	160	0.86	10.2	11.15	0.49	4.30
PBDB-T:m-Me-ITIC-Me	-	0.95	5.6	6.27	0.37	1.97
	160	0.90	9.1	9.95	0.42	3.44
PBDB-T:m-Me-ITIC-F	-	0.81	16.9	18.31	0.48	6.57
	160	0.77	22.3	21.85	0.64	10.99
PBDB-T:m-Me-ITIC-Cl	-	0.80	17.0	18.40	0.48	6.53
	160	0.76	21.0	21.97	0.60	9.58

[a] All donor:acceptor blend ratios are 1:1 and all BHJ solutions were additive free.

Table S2. The photovoltaic parameters of the OPVs based on PBDB-T:m-Me-ITIC, PBDB-T:m-Me-ITIC-Me, m-Me-ITIC-F, and m-Me-ITIC-Cl with different donor:acceptor blend ratios under the illumination of AM 1.5G, 100 mW·cm^{-2}.

Photoactive materials	Blend ratios	V_{OC} (V)	J_{SC} (mA·cm^2)	EQE (mA·cm^2)	FF	PCE[a] (%)
	1:0.8	0.87	10.7	11.38	0.47	4.41
PBDB-T:m-Me-ITIC	1:1	0.86	10.2	11.15	0.49	4.31
	1:1.2	0.89	10.1	10.77	0.51	4.60
	1:0.8	0.90	9.3	9.92	0.42	3.53
PBDB-T:m-Me-ITIC-Me	1:1	0.90	9.1	9.95	0.42	3.46
	1:1.2	0.92	8.1	8.30	0.49	3.63
	1:0.8	0.78	20.9	21.70	0.64	10.34
PBDB-T:m-Me-ITIC-F	1:1	0.77	22.3	21.85	0.64	10.99
	1:1.2	0.78	19.4	20.31	0.66	10.09
	1:0.8	0.77	20.5	20.12	0.62	9.81
PBDB-T:m-Me-ITIC-Cl	1:1	0.76	21.0	21.97	0.60	9.61
	1:1.2	0.76	17.9	18.26	0.66	8.92

[a] All BHJ solutions were additive free and all BHJ films annealed at 160°C for 20min.

Table S3. The optimized photovoltaic performances of the OSCs based on PBDB-T:m-Me-ITIC with different additives under the illumination of AM 1.5G, 100 mW·cm^{-2}.

Photoactive materials	Additives	V_{OC} (V)	J_{SC} (mA·cm^2)	EQE (mA·cm^2)	FF	PCE[a] (%)
	No additive	0.89	10.1	10.77	0.51	4.60
	DIO 0.5 vol%	0.84	7.3	7.81	0.54	3.34
	DIO 1.0 vol%	0.87	7.7	7.32	0.50	3.36
**PBDB-T:m-Me-ITIC**	DIO 1.5 vol%	0.88	8.7	9.36	0.48	3.64
	CN 0.5 vol%	0.88	8.0	8.24	0.51	3.59
	CN 1.0 vol%	0.88	8.9	9.68	0.49	3.82
	CN 1.5 vol%	0.89	8.1	8.60	0.46	3.31

[a] All donor:acceptor blend ratios are 1:1.2 and all BHJ films annealed at 160°C for 20min.

Table S4. The optimized photovoltaic performances of the OSCs based on PBDB-T:m-Me-ITIC-Me with different additives under the illumination of AM 1.5G, 100 mW·cm^{-2}.

Photoactive materials	Additives	V_{OC} (V)	J_{SC} (mA·cm^2)	EQE (mA·cm^2)	FF	PCE[a] (%)
	No additive	0.92	8.1	8.30	0.49	3.63
	DIO 0.5 vol%	0.83	6.3	6.79	0.54	2.84
	DIO 1.0 vol%	0.90	6.0	5.96	0.51	2.75
**PBDB-T:m-Me-ITIC-Me**	DIO 1.5 vol%	0.92	7.2	7.87	0.47	3.11
	CN 0.5 vol%	0.91	6.9	6.92	0.47	2.95
	CN 1.0 vol%	0.91	5.5	5.42	0.49	2.44
	CN 1.5 vol%	0.91	6.1	6.22	0.49	2.69

[a] All donor:acceptor blend ratios are 1:1.2 and all BHJ films annealed at 160°C for 20min.

Table S5. The optimized photovoltaic performances of the OSCs based on PBDB-T:m-Me-ITIC-F with different additives under the illumination of AM 1.5G, 100 mW·cm^{-2}.

Photoactive materials	Additives	V_{OC} (V)	J_{SC} (mA·cm^2)	EQE (mA·cm^2)	FF	PCE[a] (%)
	No additive	0.77	22.3	21.85	0.64	10.99
	DIO 0.5 vol%	0.78	18.3	17.99	0.70	9.97
	DIO 1.0 vol%	0.78	16.6	15.86	0.69	8.93
**PBDB-T:m-Me-ITIC-F**	DIO 1.5 vol%	0.78	18.2	18.56	0.70	9.97
	CN 0.5 vol%	0.78	21.2	20.76	0.69	11.39
	CN 1.0 vol%	0.77	19.6	20.69	0.66	10.00
	CN 1.5 vol%	0.78	18.6	18.10	0.70	10.07

[a] All donor:acceptor blend ratios are 1:1 and all BHJ films annealed at 160℃ for 20min..

Table S6. The optimized photovoltaic performances of the OSCs based on PBDB-T:m-Me-ITIC-Cl with different additives under the illumination of AM 1.5G, 100 mW·cm^{-2}.

Photoactive materials	Additives	V_{OC} (V)	J_{SC} (mA·cm^2)	EQE (mA·cm^2)	FF	PCE[a] (%)
	No additive	0.76	21.0	21.97	0.60	9.61
	DIO 0.5 vol%	0.75	13.2	13.82	0.64	6.34
	DIO 1.0 vol%	0.76	15.7	16.12	0.68	8.14
**PBDB-T:m-Me-ITIC-Cl**	DIO 1.5 vol%	0.76	20.2	18.44	0.67	10.21
	CN 0.5 vol%	0.76	15.1	15.01	0.66	7.59
	CN 1.0 vol%	0.76	15.3	14.27	0.68	7.88
	CN 1.5 vol%	0.75	12.8	13.27	0.70	6.72

[a] All donor:acceptor blend ratios are 1:1 and all BHJ films annealed at 160℃ for 20min..

Table S7. Hole and electron mobilities of the devices based on PBDB-T and the acceptors blend films.

Photoactive materials	μ_{hole} ($\times 10^{-4} cm^2V^{-1}s^{-1}$)	$\mu_{electron}$ ($\times 10^{-4} cm^2V^{-1}s^{-1}$)	$\mu_{hole} / \mu_{electron}$
**PBDB-T:m-Me-ITIC**	4.38	5.62	0.78
**PBDB-T:m-Me-ITIC-Me**	4.46	5.53	0.81
**PBDB-T:m-Me-ITIC-F**	6.55	6.47	1.01
**PBDB-T:m-Me-ITIC-Cl**	5.17	5.61	0.92
